# Factors Affecting Leaf Selection by Foregut-fermenting Proboscis Monkeys: New Insight from *in vitro* Digestibility and Toughness of Leaves

**DOI:** 10.1038/srep42774

**Published:** 2017-02-17

**Authors:** Ikki Matsuda, Marcus Clauss, Augustine Tuuga, John Sugau, Goro Hanya, Takakazu Yumoto, Henry Bernard, Jürgen Hummel

**Affiliations:** 1 Chubu University Academy of Emerging Sciences, 1200, Matsumoto-cho, Kasugai-shi, Aichi 487-8501, Japan; 2 Wildlife Research Center of Kyoto University, 2-24 Tanaka-Sekiden-cho, Sakyo, Kyoto 606-8203, Japan; 3 Japan Monkey Centre, 26 Kanrin, Inuyama, Aichi 484-0081, Japan; 4 Institute for Tropical Biology and Conservation, Universiti Malaysia Sabah, Jalan UMS, 88400 Kota Kinabalu, Sabah, Malaysia; 5 Clinic for Zoo Animals, Exotic Pets and Wildlife, Vetsuisse Faculty, University of Zurich, Winterthurerstrasse 260, 8057 Zurich, Switzerland; 6 Sabah Wildlife Department, Sabah, Malaysia, 5th Floor, B Block, Wisma MUIS, 88100 Kota Kinabalu, Sabah, Malaysia; 7 Forest Research Center, P.O. Box 1407, 90715 Sandakan Sabah, Malaysia; 8 Primate Research Institute, Kyoto University, Kanrin, Inuyama, Aichi 484-8506, Japan; 9 Department of Animal Sciences, University of Göttingen, Kellnerweg 6, 37077 Göttingen, Germany

## Abstract

Free-living animals must make dietary choices in terms of chemical and physical properties, depending on their digestive physiology and availability of food resources. Here we comprehensively evaluated the dietary choices of proboscis monkeys (*Nasalis larvatus*) consuming young leaves. We analysed the data for leaf toughness and digestibility measured by an *in vitro* gas production method, in addition to previously reported data on nutrient composition. Leaf toughness, in general, negatively correlated with the crude protein content, one of the most important nutritional factors affecting food selection by leaf-eating primates. This result suggests that leaf toughness assessed by oral sensation might be a proximate cue for its protein content. We confirmed the importance of the leaf chemical properties in terms of preference shown by *N. larvatus*; leaves with high protein content and low neutral detergent fibre levels were preferred to those of the common plant species. We also found that these preferred leaves were less tough and more digestible than the alternatives. Our *in vitro* results also suggested that *N. larvatus* were little affected by secondary plant compounds. However, the spatial distribution pattern of plant species was the strongest factor explaining the selection of the preferred leaf species.

Understanding dietary choices made by animals is an area of continuing scientific interest; such choices affect the nutritional state of animals and determine their health and fitness. However, little information is available for dietary choices of free-ranging animals as linked to a larger variety of nutritional and physical factors evaluated simultaneously. Colobine monkeys feed on ‘difficult’ to process foods, including leaves, seeds and unripe fruit, which they process in their complex, multi-chambered stomachs, where bacteria detoxify defensive plant chemicals and digest cellulose^1^. Nutritional biology studies have revealed a trend in their food choices; they prefer foods rich in protein^2^,^3^,^4^,^5^,^6^. However, there are some folivorous primates that do not show such a strong tendency, indicating that the preference for protein depends on the overall protein availability in the environment. Such selection can be clearly demonstrated only in the environments with low average protein content^7^. Thus, the examination of not just the chemical basis of food choices but also other possible factors affecting the dietary selection of primates might help in understanding their adaptive strategy in feeding behaviour.

Since food is distributed heterogeneously in nature, food abundance/biomass is one of the important factors affecting dietary selection in many primate species^5^,^8^,^9^,^10^. This is because the time required to find and handle food is a significant cost for animals in some cases, and it has been explained using the optimal foraging model, i.e. the animals tend to maximise energy gain per unit time^11^. This model can be applied to colobine monkeys even that generally spend most of their feeding time for ubiquitous food sources such as leaves^5^,^6^.

Another possible factor affecting dietary selection is mechanical toughness^12^. Primates generally avoid tough leaves and/or tough leaf parts. This has been shown for Japanese macaque (*Macaca fuscata yakui*)^13^, howler monkeys (*Alouatta palliata*)^14^ and mountain gorillas (*Gorilla gorilla beringei*)^15^. A detailed study of Angola black and white colobus monkeys (*Colobus angolensis palliatus*)^16^ has investigated the relationships between leaf toughness and foraging efficiency. The study has shown clearly that the ingestion rates are negatively correlated and masticatory investment, positively correlated with leaf toughness. This result implies that choosing leaves with a low level of toughness is an adaptive strategy of leaf-eating primates. The toughness of plant tissues depends on the amount of cell wall materials, such as cellulose, hemicellulose and lignin, i.e. the NDF^17^,^18^. If the toughness served as a proximate cue for the digestibility of plant food by primates, it would be as important in the food selection process as the nutritional properties of the food.

Another critical characteristic of primate food is its digestibility. In theory, digestibility measurements should be an optimal way to quantify the leaf quality represented by multivariate chemical factors. In the studies of primates, *in vitro* digestibility has often been measured using assays combining acid and enzymatic treatments^19^,^20^,^21^,^22^. Alternatively, the *in vitro* digestibility can be measured using an inoculum source that provides a microbiome simulating microbial fermentation. In primate studies, this assay has been performed using the faeces as inoculum, often for comparisons between different species^23^,^24^,^25^,^26^. However, the most common *in vitro* method for such measurements for herbivores uses a standardized inoculum, mostly rumen fluid of domestic ruminants (e.g. the modified Hohenheim gas test^27^). The method has been used in *in vitro* experiments examining the digestibility of leaves by herbivores^28^ and to analyse the diet of colobine monkeys^29^,^30^. Here, we applied this technique to plants potentially utilised as food by the proboscis monkeys (*Nasalis larvatus*).

*N. larvatus* is the largest foregut-fermenting colobine. Their diet consists of various proportions of leaves, fruits and flowers^31^,^32^,^33^,^34^. They are endemic to Borneo and inhabit mangroves, peat swamps and riverine forests. All previous reports show a consistent preference for leaves with high protein content^6^,^33^,^35^. However, within the preferred species, beyond the advantage of consuming the high-protein leaves, more abundant plant species are chosen, probably to maximise energy gain per unit time^6^. Yet, there are no comprehensive assessments of leaf selection in terms of chemical properties, toughness and digestibility.

Here, we added the data for leaf toughness and digestibility, measured using an *in vitro* gas production method, to the data on nutrient composition of the same plant samples obtained by Matsuda, *et al*.^6^. Using this dataset, we analysed the diet selection by *N. larvatus* on the basis of complex, integrated, multiple criteria, including the classic optimal foraging models (food abundance), chemical content, leaf toughness and digestibility. Regarding leaf selection between preferred and common plant species by *N. larvatus*, we expected to find that they would prefer leaves of better quality (high protein level, low level of toughness and high digestibility) than those of the common plant species. However, apart from the food with nutritional, mechanical and digestibility advantages, within their preferred plant species, *N. larvatus* choose the abundant leaf species. This allows them to save on travelling costs in their search for food (optimal foraging strategy is the highest priority). Consuming the abundant plant species is a prominent feature of their feeding ecology across seasons and different sites^31^,^32^. In the analysis of our comprehensive dataset, we also tried to establish which measurements might be the most relevant proxies for diet selection by this species at this particular location.

## Results

### Factors affecting leaf toughness and digestibility

The best-fit model to explain the leaf toughness, which was evaluated using the AICc criterion, included CP (crude protein) and CL (crude lipid) (Table 1), although the *Δ*AICc value of some other models was also <2.0; leaf toughness increased with the increasing Cl and decreasing CP and NDF (neutral detergent fibre) content.

**Figure 1:**
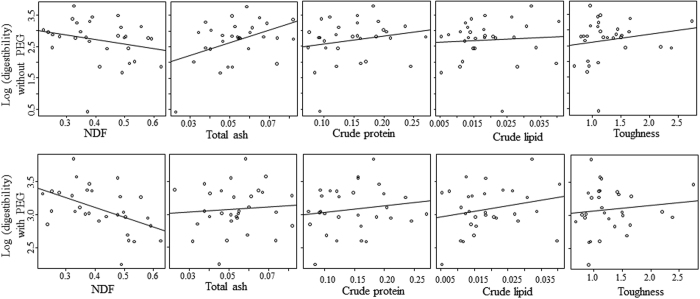
Relationship between chemical properties (values are the proportion of dry weight)/toughness (N) and digestibility (log-transformed) of leaves (ml per 200 mg DM) without PEG (top) and with PEG (bottom).

All seven models of the effects on digestibility without PEG (polyethylene glycol), with *Δ*AICc <2, included TA (total ash) with positive effects (Table 2A), positive relationships for CP and toughness and a negative one for NDF (Fig. 1). However, all the seven models of the effects on digestibility with PEG, with *Δ*AICc <2 (Table 2B), included NDF, which had a negative effect. All the other explanatory variables had positive effects on the leaf digestibility with PEG, except for toughness (Fig. 1).

### Choice between preferred and common species

**Figure 2:**
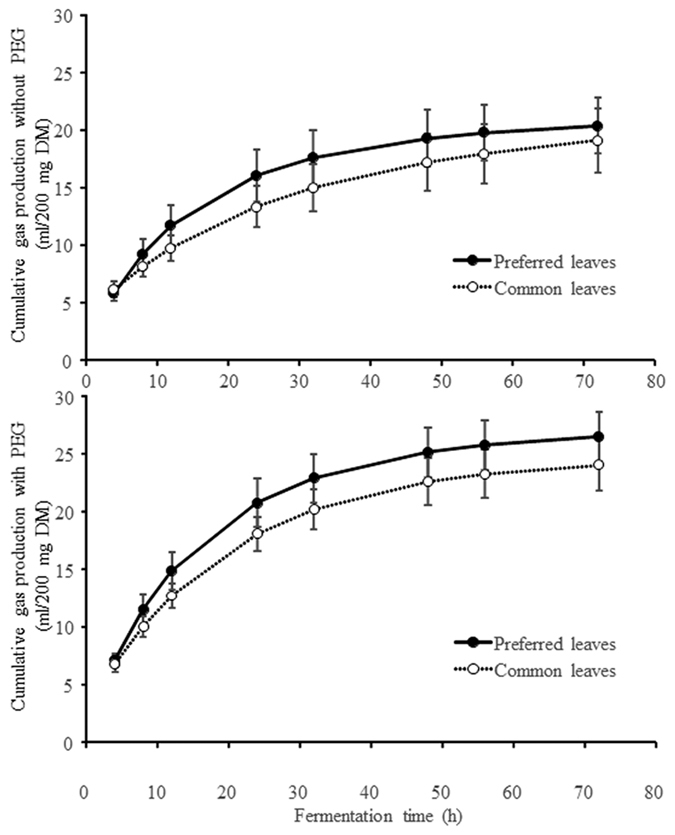
Fermentation characteristics of preferred and common plant leaves (error bar = standard error of the mean) in assays without (top) or with (bottom) the addition of PEG to neutralise the effect of tannins on the inoculum microbiome.

**Figure 3:**
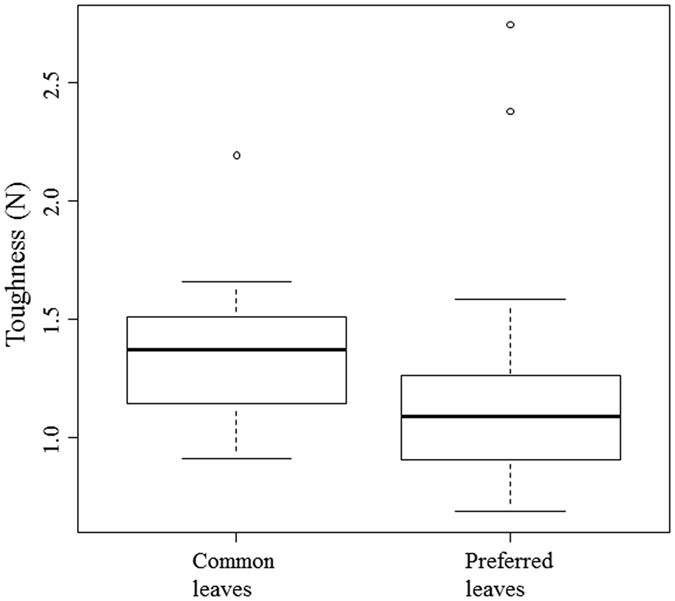
Comparison of the toughness of the common leaves and young leaves preferred by *N. larvatus*. Bold lines indicate the median. Boxes represent the range from 25 to 75% quartiles. Paired extensions show the maximum and minimum values. Outliers are indicated by circles.

Properties of common and preferred leaves are shown in Table 3. The preferred leaves generally produced more gas than the common leaves, with more consistent differences between the *in vitro* assays with and without PEG (Fig. 2). The preferred leaves were also less tough (Fig. 3 or see more detailed results in Appendix I).

As shown in Table 4, the chemical properties, and especially CP (positive effect), were more useful than other factors in the predictions of preference for young leaves. In other words, *N. larvatus* selectively fed on young leaves with high CP content, as has been shown previously^6^. However, the *Δ*AIC values of some other models were also quite small (i.e. <2.0), suggesting that other factors like the digestibility (positive effect) and leaf toughness (negative effect) can still be considered useful factors in predictions of leaf preference. For such predictions, the digestibility tested with PEG was more useful than digestibility without PEG.

The fact that the plant species abundance was always correlated negatively with the preference supports the observation that the *N. larvatus* does not feed opportunistically but preferentially select certain foods.

### Selection within the preferred plant species

**Figure 4:**
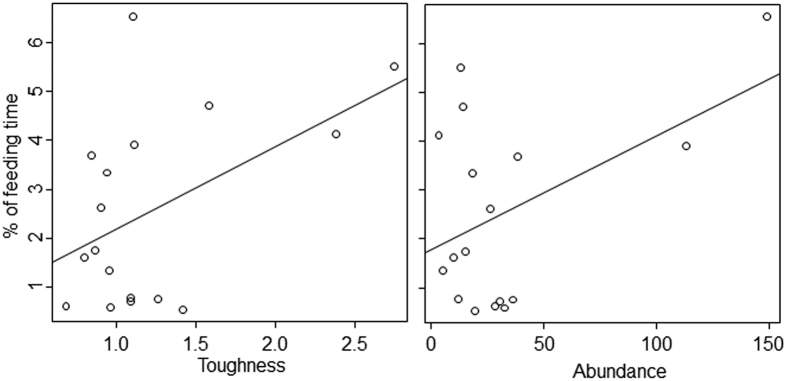
Relationship between the abundance and leaf toughness (left) and between the abundance and time spent feeding (%) on young leaves (right). Modified figure from Matsuda, *et al*.^6^

The GLMs predicting the percentages of the time spent by *N. larvatus* eating young leaves of the preferred species indicated that the leaf toughness was the factor most clearly related to this measure (Table 5). However, the trend was unexpected: the animals spent more time eating tougher leaves. Similarly, more time was spent feeding on leaves with more NDF and low digestibility than eating the other types of leaves. In contrast to the choice between common and preferred leaves, the positive relationship of the percentages of the time spent by *N. larvatus* eating each plant species of young leaves with abundance showed that, within their preferred plant species, *N. larvatus* fed opportunistically, not selectively. Nonetheless, these tendencies seemed to be driven by the two “super-abundant” plant species (Fig. 4), i.e. *Mallotus muticus* (Euphorbiaceae) and *Lophopyxis maingayi* (Lophopyxidaceae), as described in Matsuda, *et al*.^6^. Other factors were also selected in several models, indicating that the monkeys spent more time eating young leaves containing low levels of CP and CL compounds but with a high content of TA compounds.

## Discussion

We analysed the mechanisms of food selection by a leaf-eating primate, *N. larvatus*. We considered not only the chemical properties and abundance of leaves but also the toughness and digestibility which are rarely taken into account.

We found that the toughness of young leaves was negatively correlated with the CP content, one of the most important nutritional factors affecting leaf selection by colobine monkeys e.g.^2^,^3^,^5^,^6^. This indicates that the leaf toughness, assessed by oral sensation, arising from mechanical properties^17^,^36^, might be a proximate cue for the protein content for leaf-eating primates. We did not examine the effect of tannins on the leaf toughness. Toughness and chemical toxicity increase as leaves age^37^, suggesting that toughness is a cue to reduce tannin intake, which negatively affects digestion. The toughness of plant tissues depends on relative amounts of cell wall components, i.e. NDF^17^,^18^. However, we detected a negative correlation between the fibre content (NDF) and toughness. The relationship between toughness and NDF content varies for different tissues, such as the midribs, petioles and laminae^13^,^18^. Kitajima, *et al*.^38^ have shown that leaf toughness increases with increasing total bulk density and cellulose fraction, but decreases with the increasing hemicellulose and lignin content. These results suggest that we should not simply use the NDF as an indicator of toughness. The positive association between toughness and crude lipids levels might be caused by particularly thick, wax-covered cuticles in tough leaves^39^; this relationship has rarely been investigated and deserves some future attention.

Young leaves preferred by *N. larvatus* contained more protein and less NDF than those of the common plant species, as previously reported by Matsuda, *et al*.^6^. Here, we found that those preferred leaves were less tough and more digestible than those of the common plants. Leaf toughness critically affects the feeding behaviour of colobines: ingestion rates (g/min) are negatively correlated, while masticatory investment (chews/g) is positively correlated with this trait^16^. Therefore, choosing young, tender leaves would be a reasonable strategy; it would increase the consumption of superior quality food with high protein content and, at the same time, ensure efficient ingestion. Nonetheless, if the toughness were the only proximate cue for the selection of young leaves, it would be difficult to select leaves with reduced NDF content. We found a negative correlation between fibre (NDF) and toughness, and a positive correlation between digestibility and toughness. These results suggest that *N. larvatus* may use another sense, such as vision^17^, to select good quality young leaves with high protein and low fibre content. As there is a variation in the colour of young leaves (e.g. light green and red), primates may account not only for leaf toughness but also leaf colour^40^,^41^. The leaf-colour factor should be included in the future investigations to give us a more comprehensive understanding of the dietary selection of leaf-eating primates.

The results of the digestibility tests with PEG were more useful for explaining the preferences of *N. larvatus* than the tests without PEG. This might indicate that these animals are capable of neutralising the tannins. Tannins in colobine forage significantly affect *in vitro* digestibility measured using rumen inoculum^29^. One typical strategy to improve digestibility is the production of tannin-binding salivary proteins; in ruminants, it is apparently linked to particularly large salivary glands^42^. Given that colobines, in general, have large salivary glands—an often-repeated statement based on the work of Kay, *et al*.^43^—they might use a similar strategy; however, this awaits further corroboration. Indeed, *N. larvatus* does not avoid young leaves of tannin-binding species^6^, which supports this assumption. There are many reports that tannin compounds do not affect leaf selection. This trend was found in colobine monkeys including *N. larvatus* as well as other non-human primates^5^,^30^,^44^,^45^,^46^,^47^,^48^. Tannin-binding salivary proteins have been demonstrated in some primate species^23^,^49^.

We found that the most important factor affecting leaf selection among preferred young leaves was abundance, as shown by Matsuda, *et al*.^6^. In other words, *N. larvatus* was not highly selective in terms of leaf nutrition, toughness and digestibility; probably because of the relatively high quality of their preferred leaves in the forest areas adjacent to rivers. Forest areas near rivers might produce better leaves, with higher protein and lower fibre content than the primary inland forests due to the different soils and plant life histories^6^,^50^,^51^,^52^. Apart from its nutritional, mechanical and digestive properties, abundance of food is particularly important and this may be because the foraging cost saved by selecting common plants exceeds the benefit gained by selecting better quality leaves.

Our results show that the inclusion of more comprehensive measurements, beyond the quantification of crude nutrients, such as the leaf toughness or the *in vitro* digestibility, do not necessarily lead to a better understanding of dietary choices. This is an important conclusion for studies of primate diet selection, especially for comparisons between preferred and common leaf selections. CP remains probably the most easy-to-measure chemical proxy for the preferred diet items^7^. It should be noted, however, that available protein, i.e. CP minus the fibre-bound protein, may be the more relevant measure and could, in theory, yield better results. Because available protein and CP are generally positively correlated^53^, the use of CP appears nevertheless justified. However, when using the CP quantification, it is important to remember that it is not just a nitrogen-delivering nutrient. We should consider its relationships with other plant properties, including those related to the leaf age. The CP assays help to explain dietary choices as well as a more comprehensive set of predictors shown in Table 3. However, these results should not be interpreted as an indication that the other measures are not important, either conceptually or physiologically. As the crude protein content is directly related to these factors, it remains a simple proxy for a complex phenomenon. Nonetheless, selection among preferred leaf species was rather difficult to explain based on such factors. Instead, the spatial distribution pattern of certain plant species, measured as abundance, was the strongest explanatory factor for diet choices of the *N. larvatus* at this location^5^,^6^.

## Methods

### Study site and animals

The field work was conducted in compliance with animal care regulations and applicable Malaysian laws. From May 2005 to 2006, we followed a well-habituated identifiable one-male, multi-female group (one adult male, six adult females and nine immature individuals) of *N. larvatus*. The observations were conducted for 3,507 h (male: 1968 h; females: 1539 h) using focal animal sampling^6^,^31^. Continuous observations allowed the calculation of time budgets of adult monkeys, such as the proportion of the day spent feeding, and time spent feeding on individual food items. Overall, *N. larvatus* of the study group devoted 66% of feeding time to young leaves, 26% to fruits and 8% to flowers. The numbers of plant species providing young leaves, fruits and flowers were 182, 49 and 28, respectively^31^.

### Sample collection and trait measurements

We compared the chemical properties of young leaves preferred by *N. larvatus* and those of the common species in the study site. We had planned to sample the young leaves of the top 25 preferred species (defined on the basis of the proportion of total feeding time)^6^ and the 25 most abundant plant species (defined as the proportion of the total number of plant species in the study area during a vegetation survey). The survey area covered 2.15 ha including 1,645 trees and 497 vines with 180 species, 124 genera, 46 families^31^. Due to the logistical difficulties of leaf sampling from the treetops, only 17 of the top 25 preferred species and 21 of the 25 most abundant plant species could be collected. Eight plant species overlapped the two categories; thus, 30 plant species were used in this study. Samples were dried at 60 °C and milled for chemical analysis. The following nutritional components were analysed using standard procedures^54^: crude protein (AOAC no. 977.02), crude lipid (AOAC no. 963.15), total ash (AOAC no. 942.05), NDF (expressed without residual ash) (AOAC no. 2002.04). The results of these analyses were reported in Matsuda *et al*. (2013).

Three tests in leaf toughness studies have commonly been used, i.e. shearing test: measuring the work to traverse a leaf; punch test: measuring the maximum force to punch out the leaf lamina; tearing test: measuring the maximum force to tear a leaf strip^55^. Those completely different methods can apparently be used for a broad comparative survey of the toughness of primate foods; however, for some specific questions like discerning the mechanisms by which plant tissues resist fracture under incisal or molar loading, the choice of a particular test is of relevance^56^. From September 2014 to June 2015, young leaves of the same plant species were collected to assess the toughness of the leaves via punch test (originally measured as kilogram-force; 1 kgf = 1 kg × 1 G = 1 kg × 9.80665 m/s^2^ = 9.80665 N, to convert SI units to kPa). It was determined using the mass needed to penetrate a leaf, employing a penetrometer^57^,^58^ with a column of 3 mm in diameter (digital force gauges: Imada Co., Ltd, Aichi Japan). The measurement was performed for each of the 30 leaves per plant species (collected from at least four individual plants in the vegetation survey area) and the results were averaged for each species.

### *In vitro* fermentation

All experimental methods were carried out in accordance with relevant guidelines and regulations. A modified Hohenheim gas test^27^ was used in an *in vitro* fermentation system to quantify degradability of the young leaf samples (previously submitted to chemical analysis). The inoculum was obtained from the rumen fluid of cattle fed a standardized diet. An inoculum typical for *N. larvatus* was not available. However, the use of a standardized inoculum source makes it possible to compare the results with *in vitro* results from other studies. The relative abundance of microbes and their taxonomic assignments in the forestomach of the snub-nosed monkey (*Rhinopithecus roxellana*)— one of the species phylogenetically closest to *N. larvatus*—are rather similar to those in the cattle, in contrast with the microbiome of the humans or the giant panda (*Ailuropoda melanoleuca*)^59^. Two hundred milligrams of milled plant tissue was weighed in airtight glass syringes together with the inoculum, as described previously^28^,^60^, and incubated at 39 °C for 72 h. Gas production (Gp) was recorded after 4, 8, 12, 24, 32, 48, 56 and 72 h. The gas produced during the fermentation reflects the extent of food degradation. It consists of nearly equal parts of the waste gases of fermentation and the CO_2_ from the buffer (bicarbonate) reaction with the volatile fatty acids produced during the fermentation^61^. The leaves were analysed without and with 200 mg of polyethylene glycol (PEG) added to reduce the negative effects of tannins on the digestion (Makkar *et al*. 1995). Most of the samples were used in two tests on two different days (with two replicates each time). Four plant species were tested on two different days without replicates due to an insufficient size of the sample. The fermentation parameters, maximal degradation and relative degradation rate (the proportion of maximal gas production; % h^−1^), were obtained according to Blümmel and Becker^62^, via nonlinear regression using the equation


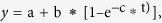


where a and b were constants (a + b = maximal gas production) [mL 200 mg^−1^ DM]; c = relative gas production rate [h^−1^] and t = time after the start of fermentation [h]. The data for only one plant species, *Vatica rassak* (Dipterocarpaceae), and only for the samples without PEG, did not fit the above regression, probably due to the strong effect of tannin. For these samples, we used several polynomial regression models comparing the AIC (the Akaike information criterion) and applied the best-fit model to estimate the fermentation parameters. We defined the estimated Gp at 40 h as the digestibility value for young leaves for *N. larvatus*; the mean retention time for different types of markers in the whole digestive tract of this species has been recently reported as approximately 40 h^63^.

### Data analysis

A linear model was used to establish whether the leaf toughness in the tested plants (30 plant species) was affected by chemical factors like NDF, crude protein, crude lipid and total ash. The measured values of toughness were log-transformed to achieve normally distributed response variables. The other factors were treated as explanatory variables. The variance inflation factors (VIFs) were smaller than the cut-off value, i.e. 10^64^. Therefore, collinearity of the explanatory variables did not affect the results. We examined a set of models with all possible combinations of the explanatory variables and ranked them using the corrected version of the AIC for small sample size, the AICc^65^. Following guidelines published for wildlife research, we selected as the best-supported models those with a *Δ*AIC(c) score ≤ 2, where *Δ*AIC(c) = AIC(c) - minimum AIC(c) within the candidate model set^65^. In other words, if AIC(c) in a model is less than 2 units larger than in the best model, it must also be discussed and reported.

A linear model was also used to find out whether the leaf digestibility (Gp at 40 h) for all collected plants, i.e. 30 plant species, was affected by leaf traits such as their chemical properties and toughness. The digestibility (with and without PEG) of young leaves was log-transformed to achieve normally distributed response variables. The other factors were treated as explanatory variables. The values of the VIFs were smaller than the cut-off value. We examined a set of models with all possible combinations of the explanatory variables and ranked them using the corrected version of the AIC.

To see which factors might explain *N. larvatus* preferences, we employed generalised linear models (GLM) using the leaf chemical properties, toughness, abundance and digestibility (with and without PEG), applying the binomial regression family calculations to obtain the AIC. The leaf preference, i.e. the preferred or common types (excluding species listed as preferred young leaf species), was treated as the categorical response variable, and the other leaf traits were treated as explanatory variables. The VIFs were smaller than the cut-off value even including the explanatory variables for digestibility with and without PEG. We examined a set of models with all possible combinations of the explanatory variables and ranked them using the corrected version of the AIC.

We also investigated the effects of leaf traits of the preferred plant species on the percentage of feeding time [as determined by Matsuda, *et al*.^6^], using GLM. The gamma family (link function: inverse, i.e. the calculated coefficient value reflects the inverse effect) was used to calculate the AIC. GLM including the explanatory variables for digestibility with and without PEG was run separately as the VIFs for these two variables were larger than the cut-off value. We examined a set of models with all possible combinations of the explanatory variables and ranked them using the corrected version of the AIC. All analyses were performed using R ver. 3.1.0^66^, employing the dredge function in the MuMIn package, ver. 1.9.13^67^.

## Additional Information

**How to cite this article:** Matsuda, I. *et al*. Factors Affecting Leaf Selection by Foregut-fermenting Proboscis Monkeys: New Insight from *in vitro* Digestibility and Toughness of Leaves. *Sci. Rep.* **7**, 42774; doi: 10.1038/srep42774 (2017).

**Publisher's note:** Springer Nature remains neutral with regard to jurisdictional claims in published maps and institutional affiliations.

## Supplementary Material

Supplementary Appendix I

## Figures and Tables

**Table 1:** Summary of model selection using linear models to investigate whether the leaf toughness was affected by chemical properties (only the models with *Δ*AICc score ≤ 2 are shown).

Intercept	Total ash	Crude lipid	NDF	Crude protein	df	Log-likelihood	AICc	*Δ*-AICc	AICc weight
0.42		11.74		−3.035	4	−4.68	19.0	0	0.25
0.53				−2.254	3	−6.49	19.9	0.94	0.15
0.62		11.48	−0.56	−2.81	5	−3.96	20.4	1.45	0.12

**Table 2:** Summary of model selection using linear models to investigate whether the leaf digestibility (Gp at 40 h) with PEG (A) and without PEG (B) was affected by several leaf traits such as chemical properties and toughness (only the models with ΔAICc score ≤ 2 are shown).

	Intercept	Total ash	Crude lipid	NDF	Crude protein	Toughness	df	Log-likelihood	AICc	*Δ*-AICc	AICc weight
(A)											
	2.31	23.2		−2.06			4	−24.6	58.9	0	0.163
	1.08	25.2		−1.98	3.83	0.41	6	−21.7	59	0.09	0.155
	2.00	22.8		−2.30	2.92		5	−23.5	59.4	0.57	0.123
	1.71	25.0		−1.78		0.30	5	−23.7	59.9	1.05	0.096
	0.97	22.8				0.40	4	−25.5	60.6	1.72	0.069
	0.34	22.7			3.36	0.51	5	−24.1	60.7	1.84	0.065
	1.64	19.8					3	−27.0	60.9	1.98	0.060
(B)											
	3.71			−1.50			3	−5.88	18.7	0	0.16
	3.55		9.07	−1.53			4	−4.60	18.8	0.11	0.15
	3.52			−1.64	1.63		4	−4.61	18.8	0.13	0.15
	3.33	4.87	9.53	−1.66			5	−3.72	19.9	1.26	0.08
	3.52	4.47		−1.62			4	−5.21	20.0	1.32	0.08
	3.35	4.27		−1.75	1.59		5	−3.94	20.4	1.69	0.07
	3.46		6.61	−1.62	1.18		5	−3.99	20.5	1.78	0.06

**Table 3:** Comparison of leaf traits and digestibility [mean ± SD] of the young leaves among abundant leaves in the study site and leaves preferred by *N. larvatus*.

	Toughness	Gp (40 h) without PEG	Gp (40 h) with PEG	Abundance	NDF	Crude protein	Total ash	Crude lipid
	(N)	(ml per 200 mg DM)		(Number of plants in survey area)	(proportion of dry weight)			
Common leaves (N = 13)	1.36 ± 0.35	16.3 ± 8.7	21.7 ± 6.8	54.4 ± 28.7	0.40 ± 0.12	0.13 ± 0.05	0.05 ± 0.02	0.02 ± 0.01
(Range)	(0.91–2.20)	(7.54–29.2)	(9.45–35.1)	(28–111)	(0.22–0.59)	(0.08–0.17)	(0.02–0.08)	(0.01–0.02)
Preferred leaves (N = 17)	1.22 ± 0.56	18.5 ± 9.8	24.3 ± 8.8	33.0 ± 38.8	0.43 ± 0.11	0.16 ± 0.05	0.06 ± 0.01	0.02 ± 0.01
(Range)	(0.68–2.38)	(6.41–44.2)	(13.5–50.0)	(3–149)	(0.25–0.62)	(0.10–0.27)	(0.04–0.07)	(0.01–0.04)

**Table 4:** Summary of model selection using GLMs to examine whether *N. larvatus* chooses young leaf species on the basis of chemical properties, abundance, digestibility and toughness (only the models with ΔAIC score ≤2 are shown).

Intercept	Abundance	Crude protein	Total ash	Crude lipid	NDF	Digestibility with PEG	Digestibility without PEG	Toughness	df	Log- likelihood	AIC	*Δ*-AIC	AIC weight
−1.23	−0.02	15.9							3	−17.3	40.6	0	0.026
−3.11	−0.02	15.6	40.3						4	−16.4	40.9	0.33	0.022
−1.94		15.3							2	−18.6	41.2	0.59	0.019
1.71	−0.02			89.5				−1.57	4	−16.8	41.6	0.99	0.016
−0.55	−0.03		42.9	98.3				−1.46	5	−15.8	41.6	1.01	0.016
−1.76	−0.02	14.3		46.4					4	−16.8	41.7	1.11	0.015
−3.87	−0.03	13.5	44.0	55.2					5	−15.8	41.7	1.13	0.015
−2.43	−0.03		47.3	71.9					4	−16.9	41.7	1.14	0.015
−2.01	−0.02	15.1				0.04			4	−17.0	42.1	1.49	0.012
−0.21	−0.02	14.8						−0.61	4	−17.1	42.1	1.56	0.012
−2.08	−0.02	14.8			2.46				4	−17.1	42.1	1.57	0.012
−0.11	−0.02			65.9					3	−18.1	42.1	1.58	0.012
−0.96	−0.02		42.8						3	−18.1	42.2	1.64	0.011
1.05	−0.02								2	−19.1	42.3	1.7	0.011
0.06	−0.02	11.2		72.0				−1.26	5	−16.2	42.3	1.78	0.011
−1.44	−0.02	15.3					0.02		4	−17.2	42.4	1.87	0.010
−3.73	−0.02	14.8	38.3			0.04			5	−16.3	42.5	1.94	0.010
−4.98	−0.02	12.2			5.62	0.09			5	−16.3	42.5	1.96	0.010
−4.00	−0.02				6.80	0.11			4	−17.3	42.5	1.97	0.010

**Table 5:** Summary of model selection using GLMs to examine the effect of chemical properties, abundance, digestibility with PEG (A) and without PEG (B) and toughness of preferred plant species (young leaves) on the percentage of feeding time (only the models with ΔAIC score ≤2 are shown).

	Intercept	Abundance	Crude protein	Total ash	Crude lipid	NDF	Digestibility with/without PEG	Toughness	df	Log- likelihood	AIC	*Δ*-AIC	AIC weight
(A)													
	3.58E-05	−1.24E-07						−1.00E-05	4	−199.4	406.8	0	0.061
	2.80E-05	−1.04E-07					3.66E-07	−1.10E-05	5	−198.7	407.3	0.52	0.047
	2.58E-05	−1.31E-07	7.33E-05					−1.08E-05	5	−198.7	407.4	0.58	0.045
	7.95E-05			−7.24E-04				−1.64E-05	4	−199.8	407.6	0.81	0.04
	6.36E-05			−6.07E-04			3.93E-07	−1.60E-05	5	−198.9	407.8	0.95	0.038
	4.67E-05	−1.14E-07				−2.35E-05		−1.07E-05	5	−199.0	408.1	1.26	0.032
	3.60E-05	−1.48E-07			1.67E-04			−1.20E-05	5	−199.1	408.2	1.33	0.031
	2.82E-05	−1.43E-07			2.15E-04		3.44E-07	−1.29E-05	6	−198.2	408.5	1.62	0.027
	5.37E-05	−8.28E-08		−2.84E-04				−1.28E-05	5	−199.3	408.5	1.7	0.026
	8.95E-05			−6.75E-04		−2.82E-05		−1.65E-05	5	−199.3	408.6	1.74	0.025
	2.23E-05	−1.14E-07	5.36E-05				2.66E-07	−1.10E-05	6	−198.3	408.6	1.75	0.025
	3.63E-05	−1.22E-07	7.05E-05			−2.25E-05		−1.12E-05	6	−198.3	408.7	1.82	0.024
	5.03E-05	−1.57E-07			2.65E-04	−3.11E-05		−1.37E-05	6	−198.4	408.7	1.88	0.024
(B)													
	3.58E-05	−1.24E-07						−1.00E-05	4	−199.4	406.8	0	0.059
	6.55E-05			−6.07E-04			4.19E-07	−1.60E-05	5	−198.6	407.2	0.42	0.048
	3.04E-05	−1.03E-07					3.44E-07	−1.09E-05	5	−198.7	407.4	0.53	0.045
	2.58E-05	−1.31E-07	7.33E-05					−1.08E-05	5	−198.7	407.4	0.58	0.044
	7.95E-05			−7.24E-04				−1.64E-05	4	−199.8	407.6	0.81	0.039
	4.67E-05	−1.14E-07				−2.35.E-05		−1.07E-05	5	−199.0	408.1	1.26	0.031
	3.6E-05	−1.48E-07			1.67E-04			−1.20E-05	5	−199.1	408.2	1.33	0.03
	5.37E-05	−8.28E-08		−2.84E-04				−1.28E-05	5	−199.3	408.5	1.7	0.025
	8.95E-05			−6.75E-04		−2.82.E-05		−1.65E-05	5	−199.3	408.6	1.74	0.025
	3.63E-05	−1.22E-07	7.05E-05			−2.25.E-05		−1.12E-05	6	−198.3	408.7	1.82	0.024
	2.41E-05	−1.13E-07	5.24E-05				2.48E-07	−1.08E-05	6	−198.3	408.7	1.82	0.024
	5.03E-05	−1.57E-07			2.65E-04	−3.11.E-05		−1.37E-05	6	−198.4	408.7	1.88	0.023
	5.22E-05	−4.93E-08		−3.58E-04			3.68E-07	−1.42E-05	6	−198.4	408.8	1.96	0.022

Note that the calculated coefficient value reflects the inverse effect (see Methods).
