# Supplementary material for: Factors Affecting Leaf Selection by Foregut-fermenting Proboscis Monkeys: New Insight from in vitro Digestibility and Toughness of Leaves
Source: Sci Rep. 2017 Feb 17;7:42774. doi: 10.1038/srep42774 (PMC5314408; doi:10.1038/srep42774)
Supplement: Supplementary Appendix I [file srep42774-s1.pdf]

Factors Affecting Leaf Selection by Foregut-fermenting Proboscis Monkeys: New Insight from *in vitro* Digestibility and Toughness of Leaves

Ikki Matsuda[1,2,3,4*], Marcus Clauss[5,], Augustine Tuuga[6], John Sugau[7], Goro Hanya[8], Takakazu Yumoto[8], Henry Bernard[4], Jürgen Hummel[9]

1. Chubu University Academy of Emerging Sciences, 1200, Matsumoto-cho, Kasugai-shi, Aichi 487-8501, Japan

   Email: ikki.matsuda@gmail.com; ikki-matsuda@isc.chubu.ac.jp

   Tel: +81-568-51-1712 (ext. 8826)

   Fax: +81-568-51-8829
2. Wildlife Research Center of Kyoto University, 2-24 Tanaka-Sekiden-cho, Sakyo, Kyoto 606-8203, Japan
3. Japan Monkey Centre, 26 Kanrin, Inuyama, Aichi 484-0081, Japan
4. Institute for Tropical Biology & Conservation, Universiti Malaysia Sabah, Locked Bag 2073, 88999 Kota Kinabalu, Sabah, Malaysia
5. Clinic of Zoo Animals, Exotic Pets and Wildlife, Vetsuisse Faculty, University of Zurich, Winterthurerstrasse 260, 8057 Zurich, Switzerland
6. Sabah Wildlife Department, Sabah, Malaysia, 5th Floor, B Block, Wisma MUIS, 88100 Kota Kinabalu, Sabah, Malaysia
7. Forest Research Center, P.O. Box 1407, 90715 Sandakan Sabah, Malaysia
8. Primate Research Institute, Kyoto University, Kanrin, Inuyama, Aichi 484-8506, Japan
9. Department of Animal Sciences, University of Göttingen, Kellnerweg 6, 37077 Göttingen, Germany

* to whom correspondence should be addressed

Appendix I Summary of the chemical properties (values are a proportion of dry weight), mechanical toughness (N) and gas production for the leaves (ml/200 mg DM) in each plant species. The table also shows the abundance (number of plants found in the surveyed area, i.e. 2.15 ha) quantified by the vegetation survey, preference and eating time (s) with the percentage of the total feeding time in brackets.

*Electronic supplementary material

Species	PEG status	Type of leaf	Abundance	Eating time	Toughness	Chemical properties				Gas production							
						NDF	Protein	Ash	Lipid	4 h	8 h	12 h	24 h	32 h	48 h	56 h	72 h
Acacia borneensis	PEG	Preferred	10	39782	0.79	0.37	0.27	0.05	0.03	9.3	11.3	13.1	17.1	19.0	21.4	22.1	23.1
	Non-PEG									6.1	8.3	10.1	13.9	15.4	17.1	17.6	18.2
Albizia corniculata	PEG	Preferred	28	15516	0.68	0.41	0.24	0.05	0.03	4.9	7.4	9.5	14.3	16.6	19.5	20.5	21.7
	Non-PEG									3.5	4.6	5.3	6.2	6.4	6.5	6.5	6.5
Antidesma thwaitesianum	PEG	Common	33	-	1.19	0.50	0.09	0.07	0.02	6.8	10.1	12.7	17.8	19.7	21.6	22.0	22.5
	Non-PEG									6.1	8.0	9.7	13.3	15.0	17.1	17.8	18.6
Bauhinia diptera	PEG	Preferred	26	64880	0.91	0.28	0.15	0.07	0.01	5.8	10.5	14.3	22.4	25.8	29.7	30.8	32.1
	Non-PEG									4.9	7.0	8.9	13.2	15.2	18.0	18.9	20.1
Bridelia stipularis	PEG	Preferred	30	18059	1.09	0.39	0.11	0.06	0.03	7.1	12.9	17.3	25.0	27.5	29.7	30.2	30.6
	Non-PEG									5.2	12.6	18.0	26.8	29.5	31.7	32.1	32.4
Crudia reticulata	PEG	Common	27	-	0.92	0.53	0.17	0.03	0.01	5.4	7.3	8.8	11.7	12.7	13.8	14.0	14.3
	Non-PEG									4.8	5.4	6.0	7.3	8.0	8.9	9.2	9.6
Carallia brachiata	PEG	Preferred	13	135484	2.74	0.38	0.19	0.04	0.04	8.8	15.7	20.6	28.6	30.9	32.7	33.0	33.2
	Non-PEG									6.4	11.3	15.0	22.0	24.5	26.8	27.4	27.9
Cleistanthus myrianthus	PEG	Preferred	19	13409	1.42	0.25	0.12	0.05	0.01	8.6	12.3	15.6	23.2	26.9	31.9	33.6	36.0
	Non-PEG									8.1	9.6	10.9	14.3	16.1	19.0	20.1	21.9
Croton caudatus	PEG	Common	46	-	1.37	0.59	0.16	0.08	0.02	2.7	5.8	8.3	13.5	15.6	17.9	18.5	19.2
	Non-PEG									2.2	5.0	7.3	12.2	14.2	16.6	17.3	18.1
Cynometra ramiflora	PEG	Preferred	3	101832	2.38	0.44	0.12	0.04	0.02	6.7	9.8	12.3	16.9	18.5	20.1	20.5	20.9
	Non-PEG									5.3	6.9	8.2	10.8	11.8	12.9	13.2	13.5
Dalbergia parvifolia	PEG	Preferred	38	90863	0.84	0.50	0.19	0.05	0.02	5.3	8.2	10.6	15.7	17.9	20.5	21.3	22.2
	Non-PEG									5.5	7.6	9.5	13.6	15.4	17.7	18.4	19.3
Dillenia excelsa	PEG	Common	98	-	1.50	0.33	0.09	0.06	0.01	7.2	10.1	12.6	17.6	19.7	22.0	22.7	23.4
	Non-PEG									6.4	8.1	9.6	13.0	14.7	17.0	17.8	18.9
Dracontomelon dao	PEG	Common	15	-	1.44	0.22	0.20	0.04	0.02	6.5	10.5	13.9	21.6	25.1	29.5	30.9	32.7
	Non-PEG									8.5	9.9	11.2	15.4	18.1	23.4	26.1	31.3
Eugenia sp. 2	PEG	Common	33	-	1.64	0.24	0.08	0.03	0.01	5.7	8.2	10.3	14.5	16.2	18.1	18.6	19.2
	Non-PEG									6.5	9.0	11.0	15.3	17.0	19.1	19.6	20.3

Eugenia sp. 3	PEG	Common	28	-	2.20	0.25	0.07	0.04	0.03	7.5	10.5	13.0	17.9	19.9	22.1	22.7	23.4
	Non-PEG									6.7	7.6	8.3	10.3	11.3	13.0	13.6	14.5
Elaeocarpus nitidus	PEG	Preferred	15	43234	0.87	0.38	0.10	0.05	0.01	6.8	9.1	11.1	16.0	18.5	22.1	23.4	25.3
	Non-PEG									5.2	6.3	7.5	10.6	12.5	15.9	17.3	19.9
Entada rheedei	PEG	Preferred	32	14733	0.97	0.56	0.23	0.05	0.03	9.5	15.0	18.8	24.2	25.6	26.5	26.7	26.8
	Non-PEG									7.4	12.1	15.5	20.8	22.3	23.4	23.6	23.8
Excoecaria indica	PEG	Common	111	-	1.14	0.48	0.16	0.05	0.02	13.0	17.3	21.0	29.0	32.5	36.9	38.2	39.8
	Non-PEG									10.4	14.3	17.6	25.2	28.8	33.6	35.1	37.2
Ficus binnendijkii	PEG	Preferred	14	115877	1.58	0.51	0.10	0.06	0.01	4.6	7.0	9.0	12.6	14.0	15.3	15.6	15.9
	Non-PEG									4.0	5.6	7.0	9.8	11.0	12.3	12.6	13.0
Hydnocarpus sumatrana	PEG	Preferred	36	18958	1.26	0.58	0.21	0.05	0.02	6.4	9.4	11.7	16.0	17.5	19.0	19.4	19.7
	Non-PEG									6.6	8.7	10.4	13.8	15.1	16.5	16.9	17.3
Lophopyxis maingayi	PEG	Preferred	113	96322	1.11	0.34	0.16	0.07	0.01	6.1	13.9	19.8	30.4	33.9	37.1	37.7	38.3
	Non-PEG									4.6	10.6	15.1	22.4	24.6	26.4	26.8	27.0
Mallotus muticus	PEG	Preferred	149	161224	1.10	0.52	0.16	0.07	0.04	4.2	7.0	9.1	12.2	13.1	13.8	13.9	14.0
	Non-PEG									3.2	4.8	5.7	6.8	7.0	7.1	7.1	7.1
Margaritaria indica	PEG	Preferred	5	33497	0.96	0.33	0.18	0.06	0.03	13.7	28.3	36.6	45.3	46.5	47.1	47.1	47.1
	Non-PEG									14.2	27.7	35.5	44.2	45.7	46.3	46.4	46.4
Nauclea subdita	PEG	Common	77	-	1.14	0.35	0.20	0.06	0.01	5.6	10.2	13.7	19.6	21.5	23.1	23.4	23.7
	Non-PEG									4.5	8.3	11.3	16.6	18.4	20.1	20.5	20.8
Pternandra galeata	PEG	Common	65	-	1.66	0.32	0.10	0.08	0.01	8.7	12.3	15.4	22.0	24.8	28.2	29.2	30.4
	Non-PEG									10.3	13.7	16.7	23.2	26.1	29.8	30.9	32.4
Spatholobus macropterus	PEG	Preferred	18	82344	0.94	0.62	0.12	0.05	0.01	4.0	6.1	7.8	11.2	12.6	14.1	14.5	15.0
	Non-PEG									2.9	3.7	4.3	5.6	6.1	6.6	6.8	6.9
Vatica rassak	PEG	Common	52	-	1.11	0.37	0.09	0.02	0.02	8.5	13.0	16.8	24.3	27.3	30.6	31.5	32.5
	Non-PEG									4.1	4.0	3.6	2.4	1.8	1.2	1.0	1.8
Ventilago dichotoma	PEG	Preferred	12	19230	1.09	0.49	0.12	0.06	0.01	8.1	12.1	15.4	22.1	24.7	27.5	28.3	29.1
	Non-PEG									6.3	9.6	12.4	18.2	20.7	23.6	24.4	25.4
Vitex pinnata	PEG	Common	55	-	1.51	0.48	0.16	0.05	0.02	6.3	9.4	11.9	16.9	18.8	20.9	21.4	22.0
	Non-PEG									5.6	8.2	10.4	14.6	16.2	17.9	18.3	18.8
Xylosma sumatrana	PEG	Common	67	-	0.91	0.49	0.08	0.04	0.01	4.0	5.5	6.6	8.5	9.1	9.6	9.7	9.8
	Non-PEG									4.0	4.2	4.4	4.8	5.1	5.5	5.7	5.9
